# A comprehensive physiologically based pharmacokinetic (PBPK) model for nicotine in humans from using nicotine-containing products with different routes of exposure

**DOI:** 10.1038/s41598-022-05108-y

**Published:** 2022-01-20

**Authors:** Ali A. Rostami, Jerry L. Campbell, Yezdi B. Pithawalla, Hamideh Pourhashem, Raheema S. Muhammad-Kah, Mohamadi A. Sarkar, Jianmin Liu, Willie J. McKinney, Robinan Gentry, Maria Gogova

**Affiliations:** 1 Center for Research and Technology, Altria Client Services LLC, 601 East Jackson Street, Richmond, VA 23235 USA; 2 Ramboll, 3214 Charles B. Root Wynd, Suite 130, Raleigh, NC 27612 USA; 3 Ramboll, 3107 Armand Street, Monroe, LA 71201 USA

**Keywords:** Biophysics, Computational biology and bioinformatics

## Abstract

Physiologically based pharmacokinetic (PBPK) modeling can be a useful tool for characterizing nicotine pharmacokinetics (PK) from use of tobacco products. We expand a previously published PBPK model to simulate a nicotine PK profile, following single or multiple use of various tobacco products [cigarettes, smokeless tobacco, and electronic nicotine delivery systems, or a nicotine inhaler (NICOTROL)] The uptake route in the model was designed to allow for three uptake compartments: buccal cavity (BC), upper respiratory tract (URT) (conducting and transitional airways) and lower respiratory tract (alveolar region). Within each region, the model includes product-specific descriptions of the flux of nicotine into plasma, as well as the flux of nicotine from the BC and URT to the gastrointestinal tract. These descriptions are based on regional deposition and diffusion models of nicotine into plasma, which depends on the product type. Regional deposition flux combined with regional differences in physiological parameters (e.g., blood perfusion ratio and tissue thickness) play a key role in the product-specific PK profile of nicotine. The current model describes the slower flux of nicotine into plasma across the BC and URT, as well as the rapid flux known to occur in the alveolar region. Overall, the addition of the BC and respiratory tract compartments to the nicotine model provided simulation results that are comparable to the nicotine time-course plasma concentrations reported from clinical studies for the four product categories simulated.

## Introduction

In 2016, the United States Food and Drug Administration (FDA) finalized a rule extending the Center for Tobacco Products (CTP) regulatory authority to cover all tobacco products, including electronic nicotine delivery systems (ENDS) that meet the definition of a tobacco product^1^. As discussed in their Comprehensive Plan for Tobacco and Nicotine Regulation^1^, nicotine and the regulation of nicotine in tobacco products are at the center of the Agency’s tobacco regulation efforts. In its proposed premarket tobacco product applications (PMTA) rule^2^, FDA emphasizes the importance of understanding nicotine pharmacokinetics (PK), arising from use of a new reduced risk product, both independently and in relative comparisons to other tobacco products, such as conventional cigarettes. Among other factors, understanding how the pharmacological profile of nicotine may be impacted by product characteristics and/or use behavior, is integral to understanding the new product’s abuse liability potential and evaluating the product’s health risks. As an example, the proposed rule mentions that the pharmacological profile of nicotine may impact use behavior, which in turn may impact the overall exposure of an individual to other harmful and potentially harmful compounds (HPHCs). To enhance understanding of the concentration of nicotine in plasma resulting from the use of different new and existing tobacco product types and the impact of human variability on internal dosimetry; multiple physiologically based pharmacokinetic (PBPK) models have been developed to assist in estimating these concentrations^3–9^. Although some of these models did include different administrations routes, none of them addressed the variability in nicotine exposure and deposition patterns that would occur from using different types of nicotine containing products, ranging in exposure routes from oral delivery to inhalable aerosols. Furthermore, the current model allows simulation of product-specific uptake of nicotine across a wide range of nicotine-containing products and the corresponding permeation in a single PBPK model.

The objective of this work was to extend the previously published nicotine PBPK models^3–5^ to cover a variety of nicotine containing products and estimate the subsequent impact of using these products on nicotine PK profiles. This work allows for a combination of product-related exposure routes in a single comprehensive model for human, that includes regional absorption, followed by diffusion through the corresponding tissues and rate of transfer to the circulation system. The regional absorption [buccal cavity (BC), upper respiratory tract (URT), and lower respiratory tract (LRT)] requires allowing for anatomical and physiological differences between these regions to be captured, thereby resulting in a more realistic representation of the processes occurring in the human respiratory tract (RT). The previous models are each related to a specific route of exposure for rats, human or monkey as described below. Robinson and co-workers^4^ developed the initial nicotine PBPK model for humans using PK data in humans after intravenous infusion of nicotine. Plowchalk et al.^3^ reported a nicotine PBPK model for rats which was used to simulate intra-arterial or intravenous administration of nicotine. Teeguarden and co-workers^5^ attempted to use the available kinetic data of rats and humans to provide a formal calibration of the nicotine model to allow for other routes of exposure. Their model included simulation of kinetic data from routes of exposure other than intravenous infusion (i.e., oral exposure in rat and human and inhalation from cigarette in human); however, the intake was described as direct infusion into plasma. The mass of absorbed nicotine was estimated as part of the model calibration. One common aspect of these models was the inclusion of a pharmacodynamic component which accounted for the tissue binding of nicotine and the resulting change in cardiac output. A more recent joint publication by the FDA National Center for Toxicological Research and CTP discusses how a PBPK model with age-specific systemic clearance parameters for nicotine and cotinine, can be employed to understand differences in nicotine deposition between adult and adolescent squirrel monkeys, following intravenous administration^10^.

We have tried to eliminate the limitations of prior works by allowing for simultaneous absorption from different routes of exposure, distribution of absorptions along the entire airway, anatomical and physiological differences among absorption sites and how the diffusion through the different tissues affect PK profile. Absorption of nicotine in aerosol across different regions of the human RT mainly depends on the nicotine gas/particle partitioning, which defines the absorption/deposition sites of nicotine in the RT^11^. For conventional cigarettes, it is assumed that most of nicotine is absorbed in the LRT and it is instantly transferred to the bloodstream. These assumptions are justified because (1) most of nicotine in cigarette smoke remains in particles and (2) the surface area of the alveolar region is large and the blood-air barrier is small. Such assumptions are not necessarily valid for ENDS aerosol where higher portions of nicotine could be transferred to the upper airway surface as vapor phase. Limitations arise from the models described above because advanced knowledge of the distinct PK data is required for estimating direct intake into the blood compartment resulting from different product and usage scenarios.

It is important to expand on the previously available models to incorporate a more robust intake model for estimating nicotine uptake distribution along the BC, gastrointestinal (GI) tract, and RT from using different types of tobacco products. Depending on the product and conditions of use, nicotine is absorbed in the BC, GI, and/or RT compartments. The model presented here allows for regional deposition of nicotine in the BC, URT (conducting airways), and LRT (transitional airways and alveolar region) to be taken into account. Our hypothesis in this effort is that the differences in deposition locations across BC and the RT result in significant differences in nicotine PK from use of different product types. The difference is associated with the slower rate of nicotine transfer to the bloodstream due to higher air-blood resistance across the thicker tissues in the BC and URT, compared to the LRT. Learning from nicotine uptake will be helpful in extending the nicotine PBPK model to describe PK of other chemicals of potential concern in nicotine containing products.

## Modeling methods

**Figure 1:**
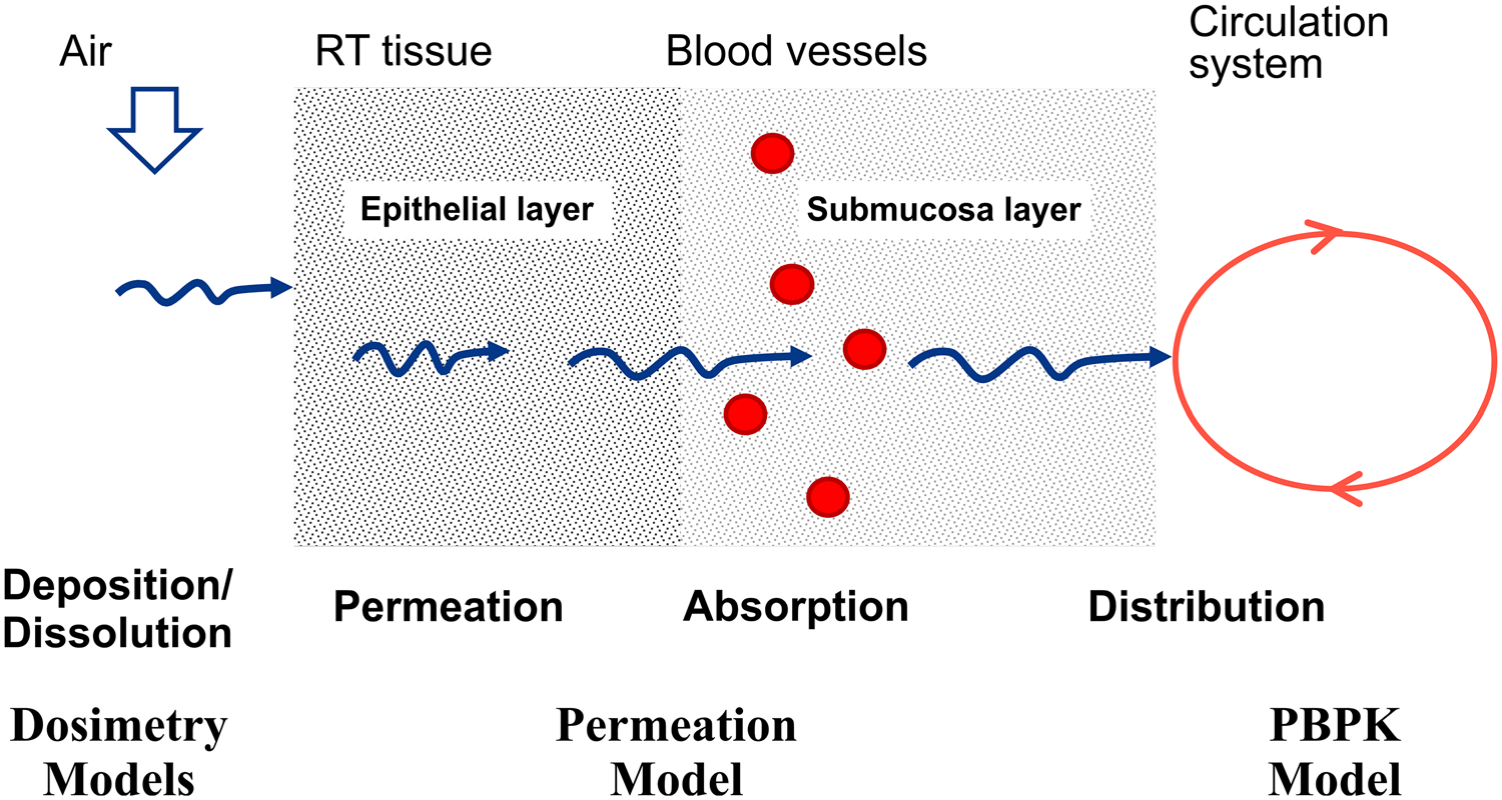
Nicotine transport process from RT airway to circulation system including permeation through RT tissues.

The main contributions of this work to the existing nicotine PBPK model are three-fold. First, nicotine dosimetry distribution (BC, GI, or other RT compartments) resulting from using different nicotine-containing products that result in different systemic routes of exposure is more realistically captured. Second, usage conditions (frequency and duration of use, intensity of use such as puff volume for aerosol inhalation or squeezing of oral products, swallowing and spitting frequency) are more accurately estimated. Third, the anatomical and physiological differences between the URT and LRT and their effects on the rate of nicotine permeation to the bloodstream are accounted for. The entire nicotine release and transfer rates consisting of several phenomena that are partially shown in Fig. 1, are mathematically represented and computationally estimated.

To incorporate these processes in the PK profile predictions, three types of models are needed: (1) Dosimetry models predict the uptake distribution of the available nicotine in the BC, GI, URT, and LRT. In PK studies, the dosimetry information is key to correlate observed clinical results with the delivered dose. The dosimetry models are primarily driven by the route of exposure, making them more dependent on product specific parameters, thereby requiring specific dosimetry models for different types of nicotine containing products. (2) Permeation model, allows for calculating the rate of nicotine transfer to the circulation system in each of the three regions, depending on the type of the product. The same permeation model is applied to all products, with differences arising primarily from anatomical parameters (e.g., tissue thickness, surface area, etc.) differing in different parts of the RT. (3) PBPK model with three distinct uptake compartments incorporates physiological process such as absorption, metabolism and clearance of nicotine to estimate distribution and uptake of nicotine into plasma. Each model is described in more detail below.

### Dosimetry models

The primary inputs to the PBPK model are the rate of nicotine transfer to the tissues at the absorption sites and duration of exposure. For oral products, nicotine is released from the product, mixed with the saliva, partially transferred to the oral tissue. For inhalable vapor or aerosols, nicotine transfers to the airway tissues via vapor phase and particulate phase in parallel. The physics associated with each and the corresponding mathematical modeling for these products are different and hence require different dosimetry models. The dosimetry models described below show how the nicotine transfer rate to the tissue is calculated for each product category.

#### Aerosol deposition model: dosimetry estimates for inhalable aerosols generated from use of ENDS and conventional cigarette products

Chemical constituents in aerosols delivered by ENDS and conventional cigarettes are present both in vapor phase in air, as well as in the liquid phase as droplets. The partitioning between the two phases depends on the volatility of constituent and what fraction of the constituents is bound in the liquid droplets. This, along with the hygroscopicity of aerosol, as well as complex aerosol composition, makes the dynamics of aerosol transport in the RT much more complex than the vapor phase alone. Furthermore, the aerosol can deposit in different locations across the RT; starting from the BC down to the alveoli. Therefore, more complex models are needed to predict the regional absorption of nicotine in the RT from products that generate inhalable aerosols. Detailed descriptions of models for characterization of deposition of aerosol constituents in the RT are beyond the scope of this article. Readers are referred to our prior publications^12,13^ for more details.

**Figure 2:**
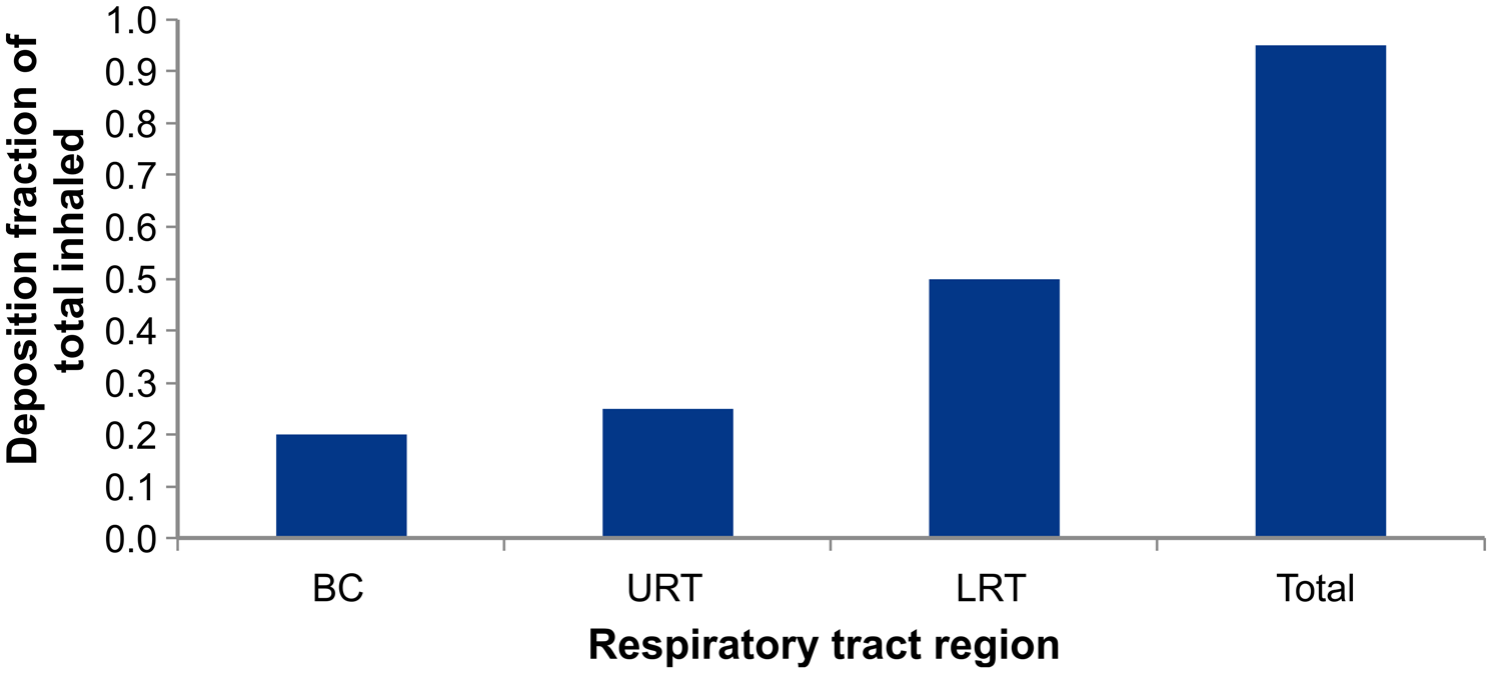
Example of regional and total nicotine absorption distribution after using an ENDS device (MT: mouth and throat, URT: upper respiratory tract, LRT: lower respiratory tract). Results are predicted by aerosol deposition model described in^12,13^.

As an example of application of the aerosol deposition model, we used data from a clinical study sponsored by Altria Client Services LLC and conducted by contract research organization Celerion, in compliance with Good Clinical Practice principles. Subjects were instructed to take 10 puffs, each 5 s duration and 30 s puff intervals on a cig-a-like ENDS device. Based on machine testing, this device delivered approximately 0.15 mg nicotine in a 5 s puff. The distribution of nicotine deposition in the RT, employing the aerosol deposition model described in our prior publications^12,13^ is shown in Fig. 2. Results show that for this use case, approximately 20% of inhaled nicotine is absorbed in the BC, 25% in the URT, 50% in the LRT and 5% is exhaled. This distribution varies considerably depending on the use topography and the pH of aerosol. The significance of pH is on the fraction of free base nicotine and vapor-particle partitioning. More free base nicotine means more volatility, hence more vapor phase.

For conventional cigarettes, 100% of inhaled nicotine is assumed to be retained in the body after each puff^14^. Furthermore, 95% of nicotine of the retained nicotine is assumed to be absorbed in the LRT and is transferred instantly to the circulation system. The remaining 5% is assumed to be absorbed in the BC and the URT.

#### Vapor absorption model: dosimetry estimates following use of products that generated inhalable nicotine vapors

The uptake of nicotine from products that deliver inhalable vapor follow a different mechanism compared to those described earlier. Inhaled vapor is transferred from air to the RT surface. In general, vapor phase nicotine, upon inhalation, is rapidly absorbed in the BC and URT. This is due to the rapid diffusion of vapors along the BC and URT. As an example of vapor absorption case, we considered a vapor inhaler product (NICOTROL). In a study conducted on this nicotine inhaler, positron emission tomography (PET) imaging was used to demonstrate that most (~ 95%) of the nicotine vapor released from an inhaler is absorbed in the BC, with ~ 5% being absorbed in the URT, and almost negligible amounts being deposited in the LRT^15^. It was also shown that about 2 mg of nicotine is systemically absorbed after taking 80 puffs on the nicotine inhaler device over 20 min^15^.

**Figure 3:**
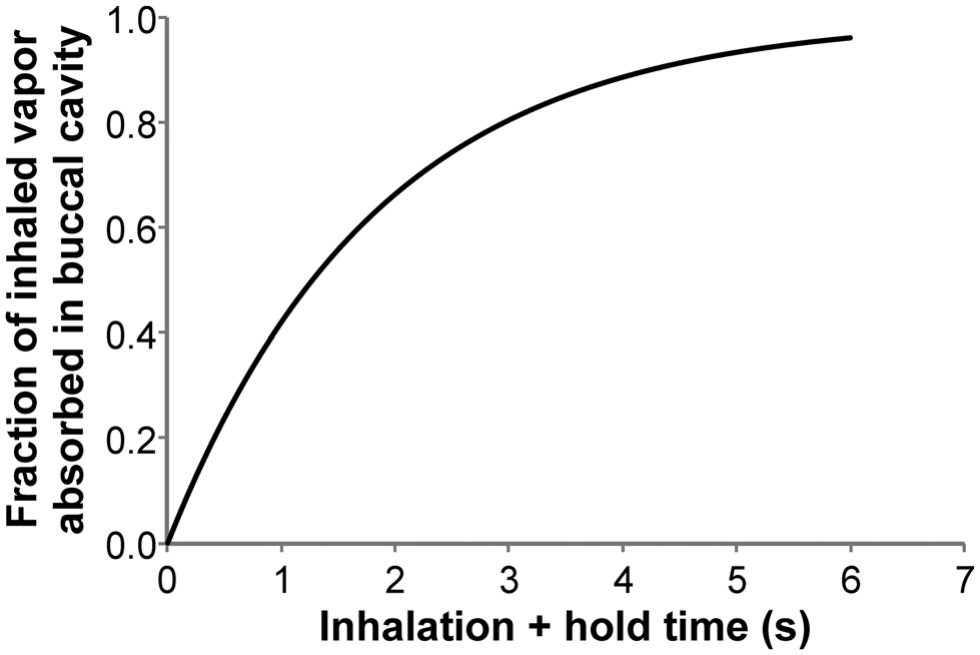
Nicotine absorption in buccal cavity, as predicted by vapor absorption-based dosimetry model (Eq. 1) as a function of total inhalation and mouth hold time.

Generally, the inhalation process involves (1) taking a puff on the device, (2) mouth hold, followed by (3) inhaling air to push the puff down in the lung. In a one-dimensional and transient approach, the whole process can be modeled either as a bolus of mixture pushed by fresh air, or as well-mixed with fresh air, or including axial convection and diffusion. Since all three approaches resulted in similar absorption rates in BC and URT, we used the well-mixed approach to estimate the rate of vapor absorption in the BC as described in Eq. (1):

1 $$ { }\frac{{C_{final} }}{{C_{initial} }} = \exp \left\{ { - \left( {\frac{{k_{g} k_{t} }}{{k_{g} + k_{t} }}{ }\frac{{A_{s} }}{V}{ }t} \right)} \right\} $$

where k_g_ and k_t_ are air-side and tissue-side mass transfer coefficients^16^, A_s_ is the BC surface area, V is the puff volume, t is the time, C is the concentration of compound in air. The time t includes both the puff inhalation time and the mouth hold time. Figure 3 shows the fraction of inhaled nicotine absorbed in the BC as a function of (puff inhalation + mouth hold) time, as predicted by Eq. (1).

#### Dissolution model: dosimetry estimates following use of oral nicotine delivery products, including smokeless tobacco

**Figure 4:**
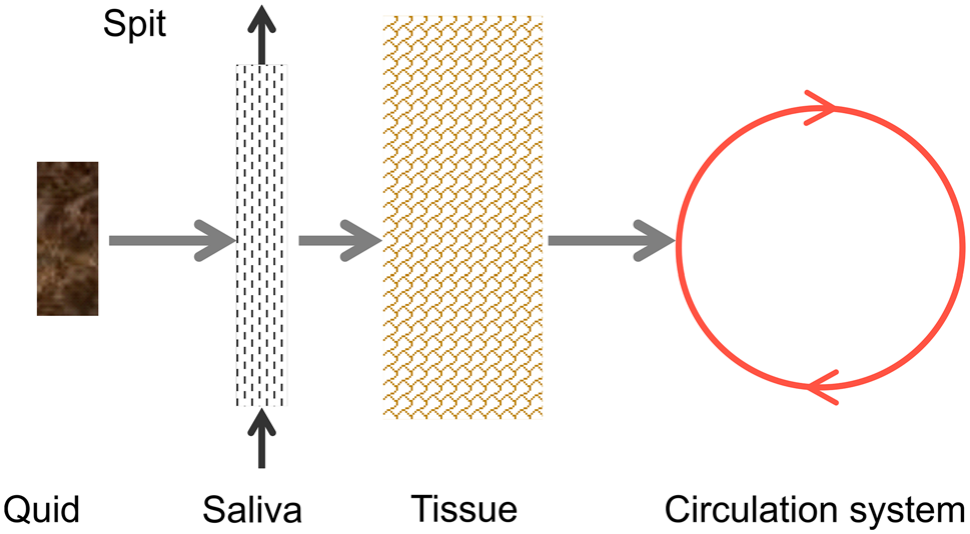
Process for release and transport of nicotine from a smokeless product, including dissolution in saliva, dilution by saliva, intermittent expectoration and or swallowing and permeation through the tissue.

For smokeless tobacco products, the major route for nicotine transfer is through oral absorption, which occurs slowly. A dissolution model has been developed to estimate the rate of release of nicotine in the BC during the use of loose smokeless tobacco products, the amount expectorated and the rate of transfer to the tissues. The mass flow path of nicotine from the oral tobacco product (used in the form of a quid placed within the BC) to the blood circulation system is depicted in Fig. 4.

**Figure 5:**
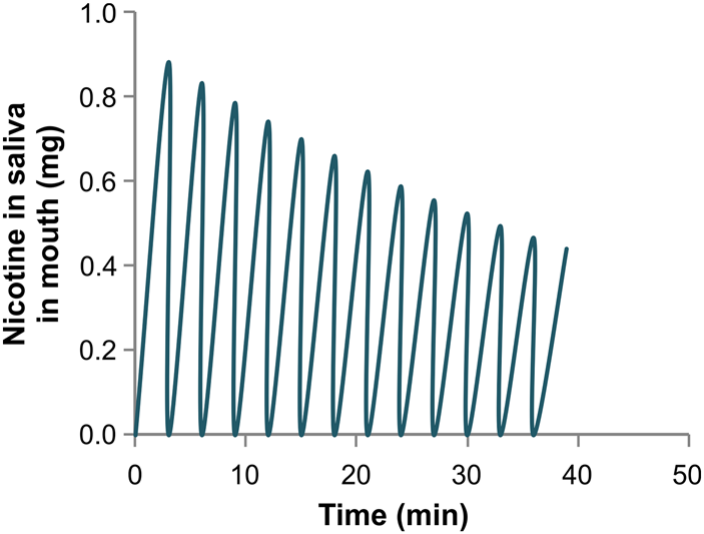
Amount of nicotine in the saliva predicted by the dissolution model over 40 min of MST use. It rises before two expectorations and rapidly drops after each expectoration.

The utility of the dissolution model is shown in the following example. In a clinical study sponsored by Altria Client Services LLC and conducted by contract research organization Celerion, in compliance with Good Clinical Practice principles, 24 subjects were instructed to use 2 g of loose moist smokeless tobacco (MST) for 40 min, during which they were allowed to spit out the saliva at arbitrary intervals, but instructed not to swallow. The amount of nicotine in unused and used quid, as well as the amount expectorated, were analytically measured and reported. Analysis of the quid post-use indicated that out of the approximately 21 mg of nicotine present in the unused quid, about 10 mg was reported to be released. Further measurements indicated that out of the 10 mg released over the use period, 75% was expectorated and only 25% absorbed in the BC. The dissolution model used these end point data from the clinical study to determine the time profile of oral nicotine absorption over the product use time. By performing an overall mass balance and considering all transfer rates, the nicotine concentration in the saliva for this example is shown in Fig. 5. Nicotine concentration in saliva increases between expectorations and drops rapidly after each expectoration, as fresh saliva is secreted. Note that though *ad-libitum* expectoration was allowed in the clinical study, due to absence of any measured data on expectoration times, it was assumed that expectorations occurred at constant time intervals every 3 min. The rate of nicotine transfer to the tissue during 40 min of MST product use can be estimated from Eq. (2):

2 $$ Q_{t} = \frac{{C_{q,0} }}{2}\left[ {1 - 2b + \left( {1 + 2b} \right)e^{ - kt} } \right]k_{t} t_{sp} $$

where Q_t_ = nicotine mass transfer to tissue (kg) between spits, $b = V_{q} /k_{qs} t_{sp}$, $C_{q,0}$ = nicotine concentration in pre-use quid (kg/m^3^), V_q_ = quid volume (m^3^), K = 0.0003179 (1/s), exponential release coefficient (1/s), k_t_ = tissue transfer rate (m^3^/s) = P_t_*A, P_t_ = permeability through tissue (m/s), K_qs_ = quid to saliva mass transfer coefficient (m/s), t_sp_ = spit interval (s).

**Figure 6:**
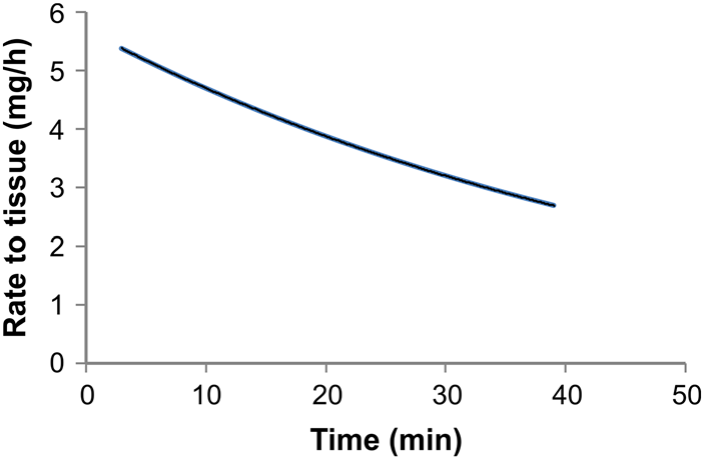
Rate of nicotine transfer from saliva to the tissue over 40 min of a smokeless tobacco use based on the dissolution dosimetry model (Eq. 2).

Figure 6 shows the rate of nicotine transfer to the tissue over the 40 min product use period, as predicted by the dissolution-based dosimetry model.

### Permeation model

Permeation model estimates the rate of nicotine transfer through the tissues as a function of time. The main inputs to this model are the flux of nicotine to the air side of the tissue, tissue thickness and the diffusion/permeation coefficient. The nicotine flux to tissue in each compartment of the airway depends on the product type and usage conditions, and as discussed in previous sections, estimated using the dosimetry models. The diffusion coefficient depends on the pH in the respiratory fluid and the tissue, which can be a function of product type, and buffering capacity. The tissue thickness is an anatomical feature and independent of the product type. Therefore, we only introduce one permeation model for all category of products. However, we picked diffusion coefficient which is representative of the pH value. In the absence of any information on the pH values in the saliva or respiratory fluid, we selected a diffusion coefficient corresponding to the physiological pH.

Once the absorption rates and distributions for different products are specified using the dosimetry modeling approaches outlined in the previous section, the rate of transfer to the bloodstream must be determined by a permeation (diffusion) model. It is expected that, for a given nicotine flux to the tissue surface on the air side, the transfer rate to plasma in the BC is much slower than in the alveoli of the LRT. Air-blood transfer barrier in the BC and to some degree in URT is much larger than that in the alveoli, because of the larger tissue thicknesses and smaller surface areas in these compartments. It is generally assumed that, due to the barrier thickness being only a few micrometers in the alveoli region, the nicotine absorbed in this region is instantly transferred to the blood and carried away by the circulation system. On the other hand, there is considerable delay in transfer of nicotine absorbed in the BC and URT to reach the circulation system.

Permeation from the RT surface to the circulation system is simply modeled as a transient diffusion process through a flat wall separating the two sides. The air side of the wall (i.e., source side) is subjected to a specified, time-dependent flux of nicotine, and the blood vessels on the other sides serve as a sink. This rate can be obtained by solving the following transport equation

3 $$ \frac{\partial C}{{\partial t}} = D\frac{{\partial^{2} C}}{{\partial x^{2} }} $$

subject to the following initial and boundary conditions:$t = 0;C\left( {x,t} \right) = 0$

$$ x = 0;D\frac{{\partial C\left( {x,t} \right)}}{\partial x} = prescribed $$

$$ x = L;C\left( {x,t} \right) = 0 $$

where *C* is concentration in tissue, *D* is the tissue diffusion coefficient, *t* is the time, *x* is dimension across the tissue, *L* is the tissue thickness. Once the concentration profile is determined, the rate of transfer to the blood vessel can be determined from $DA\frac{{\partial C\left( {x,t} \right)}}{\partial x}$|_L_ with *A* being the transfer surface area.

The main parameters that define the diffusional transfer rate are the nicotine diffusion coefficient in the tissue, tissue thickness, and transfer surface area. The diffusion coefficient is obtained from Adrian et al.^17^, and the oral mucosa thickness is obtained from Corley et al.^18,19^.

**Figure 7:**
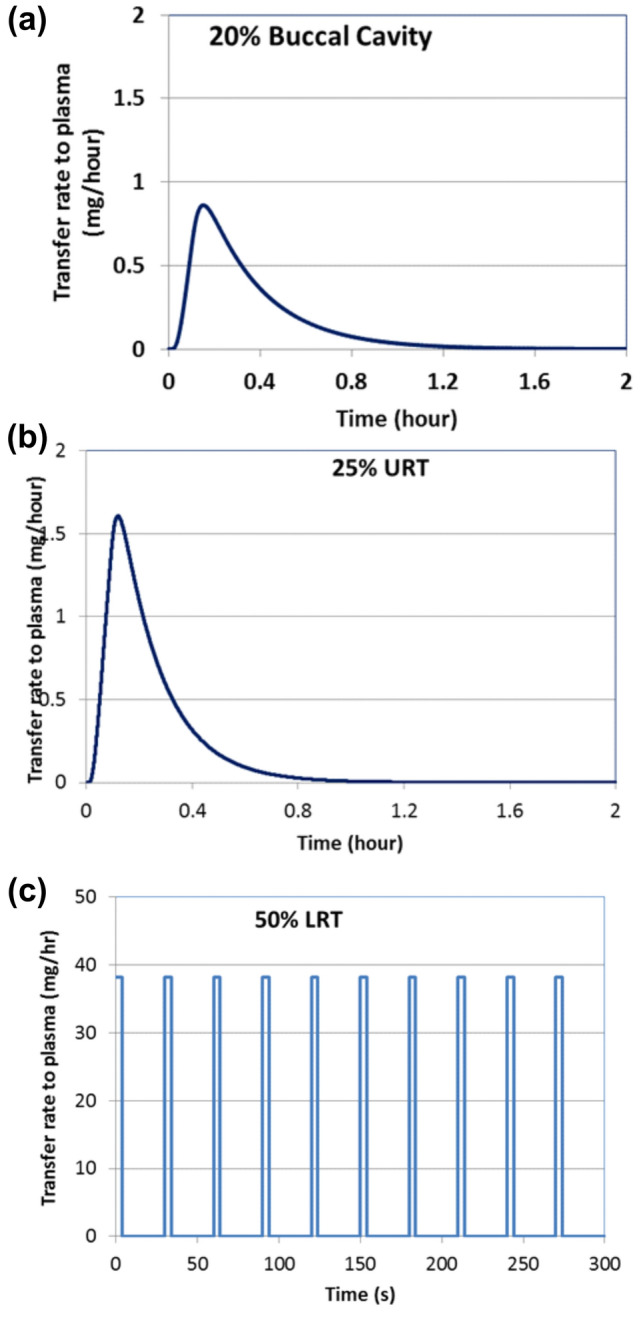
Rate of nicotine transfer to plasma from using an ENDS product with regional distribution shown in Fig. 2, calculated from permeation model. (**a**) 20% BC, (**b**) 25% URT, and (**c**) 50% LRT (5% exhaled).

**Figure 8:**
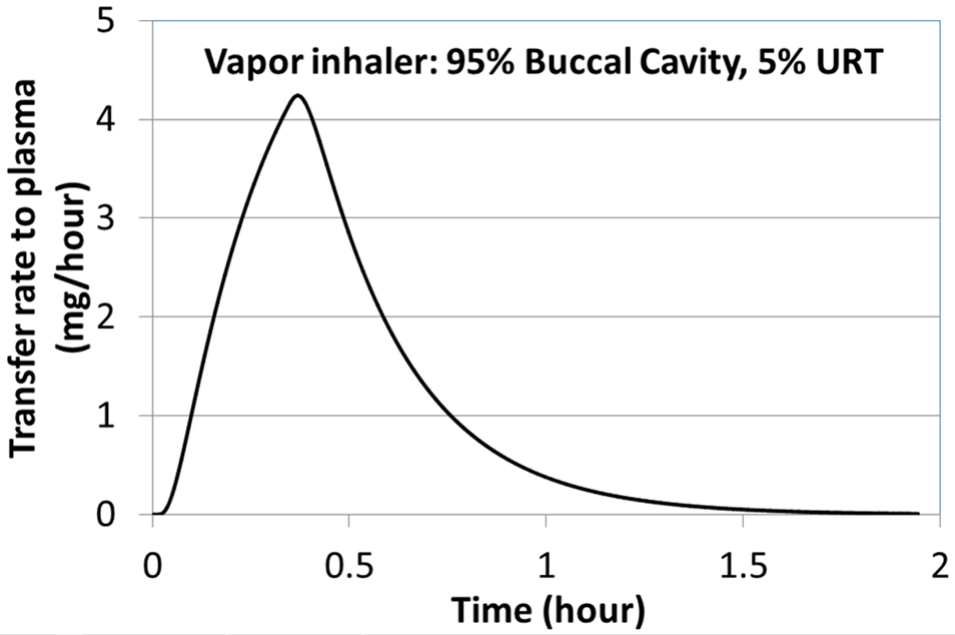
Rate of nicotine transfer to blood from using vapor inhaler with 95% BC absorption and 5% in URT calculated from permeation model based on the diffusion Eq. (3).

**Figure 9:**
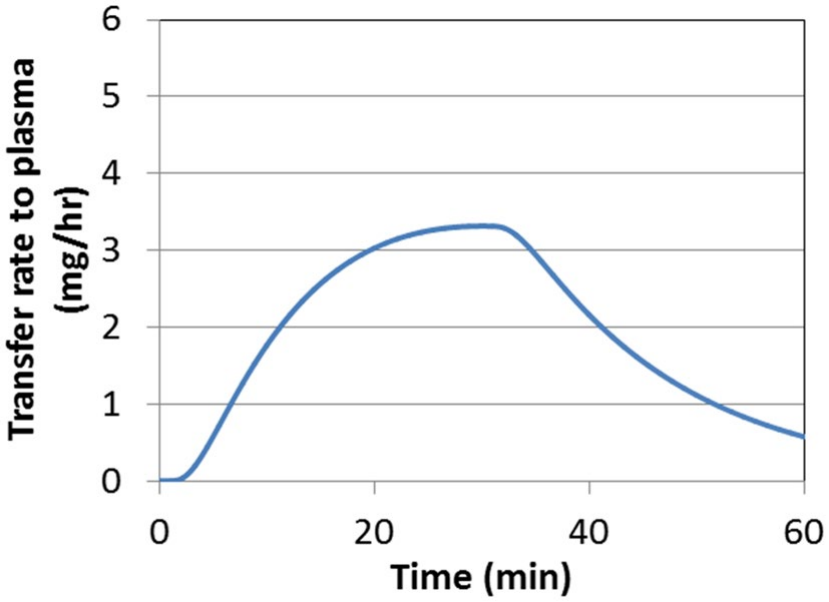
Rate of nicotine transfer to plasma in the BC from the smokeless tobacco product, corresponding to the release rate shown in Fig. 4, calculated from the permeation model.

As will be shown for specific examples presented in the Results and Discussion section, we used dosimetry models followed by the permeation model, to estimate the rate of nicotine transfer to the circulation system as a function of time for intravenous infusion and four different types of nicotine containing products: cigarettes, ENDS, inhaler and smokeless tobacco. The rate of nicotine transfer into the circulation system serves as a key input into the PBPK model. Results for four example products are summarized in Figs. 7, 8 and 9. For cigarettes, 95% of the inhaled nicotine is transferred instantaneously to the circulation system (similar to Fig. 7c), only 5% in the URT (similar to Fig. 7b). for other products, the uptake distribution are determined from dosimetry models.

### Nicotine PBPK model

#### Model structure

**Figure 10:**
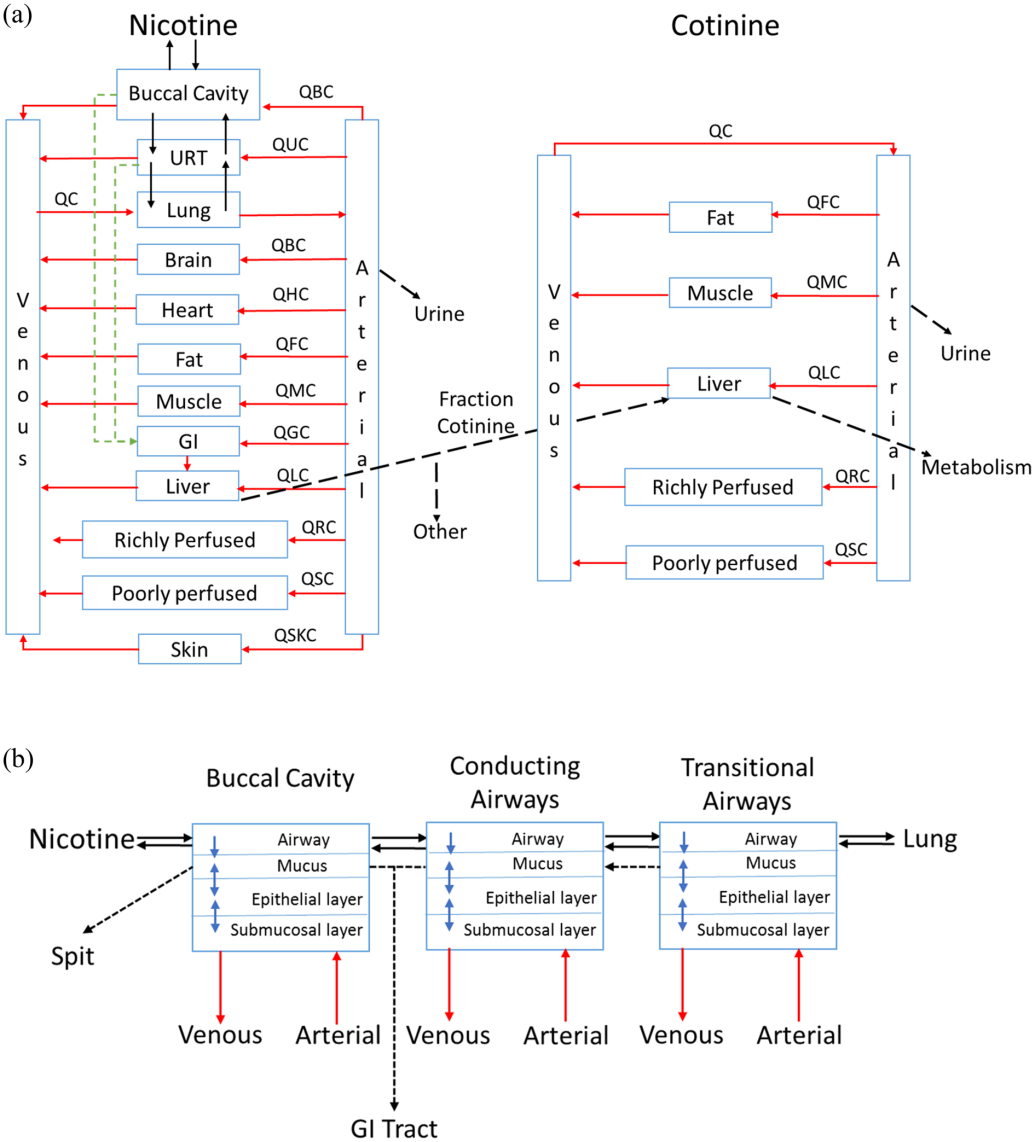
Nicotine PBPK model structure including all compartments included, with three separate administrations routes: (**a**) physiological model, (**b**) respiratory tract sub-models.

The primary goal of this effort was to incorporate tissue specific contact with nicotine after exposure to a range of products including inhaled and oral delivery. The structure of the nicotine PBPK model proposed here is shown in Fig. 10. The physiological model is based on the one described by Robinson et al.^4^ which specifies the uptake and distribution of nicotine and cotinine following exposure to nicotine in humans. Teeguarden et al.^5^ subsequently used this model to estimate uptake and clearance of nicotine and cotinine with a simplified version, which included moving metabolic clearance of nicotine to the arterial blood compartment. As such, our model parameterization will include parameters from both the above-mentioned efforts. The nicotine model of Robinson et al.^4^ described the uptake and distribution in lung, brain, heart, fat, muscle, liver and skin; along with lumped compartments for rapidly and slowly perfused tissues. Nicotine was cleared either through hepatic metabolism or through urine. The cotinine sub-model included fat, muscle, liver and lumped (slowly and richly perfused) tissue compartments. Notably, the published nicotine models simulated the exposure to nicotine as input directly as an intravenous infusion for inhaled nicotine or bolus into the oral absorption compartment. As our effort is focused on modeling nicotine PK from use of different types of nicotine containing products and the product specific dosimetry impact on nicotine PK, we expanded the nicotine PBPK model to include compartments for the BC and for the URT airway tissues (conducting and transitional airways), using the diffusion model previously used for nasal and airway tissues as described in Campbell et al.^20^ and other hybrid CFD-PBPK models^21^. This segregation of deposition compartments is necessary for accurately predicting nicotine PK from use of different types of nicotine containing products. As previously discussed, differences in surface areas, tissue thickness and air blood barrier in these areas, significantly impact the permeation rate of nicotine into the bloodstream. Another basic premise is that the compound of interest will diffuse bi-directionally between the mucus, epithelium tissue and submucosal layers. As described in previous sections, for this work, deposition of nicotine in the RT was estimated outside of the PBPK model using an aerosol deposition model for inhalable aerosol and a diffusion-based nicotine release model for oral products.

#### Model parameterization

Human physiological parameters employed within our model are shown in Table 1. Tissue and blood flow fractions for major organs were adopted from the International Commission for Radiological Protection (ICRP)^22^. The surface areas and thicknesses of the BC and RT tissues are based on information published by Corley et al.^18,19^. The chemical specific parameters for nicotine and cotinine (Table 2) were primarily based on the study by Robinson et al.^4^ with the constants for change in heart rate with nicotine exposure and oral absorption rates from Teeguarden et al.^5^. The oral absorption description for nicotine is a pseudo-physiological compartment with first-order uptake of the fraction available directly input into the liver tissue compartment.

**Table 1:** Physiological parameters for the nicotine PBPK model^5,18,19,22^.

Parameter	Label	Value	Source
Body weight (kg)	BW	73.0	Various
Cardiac output (L/h/kg BW0.75)	QCC	16	ICRP^22^
Heart rate (beats per min)	HRO	61.1	Teeguarden et al.^5^
Tissue volumes (fraction of body weight)			
Heart	VHC	0.0044	ICRP^22^
Brain	VBC	0.02	ICRP^22^
Fat	VFC	0.258	ICRP^22^
Liver	VLC	0.024	ICRP^22^
Skin	VSKC	0.042	ICRP^22^
Muscle	VMC	0.34	ICRP^22^
Arterial blood	VABC	0.02	ICRP^22^
Venous blood	VVBC	0.05	ICRP^22^
Rapidly perfused	VRC	0.03	Calculated
Slowly perfused	VSLOWC	0.08	Calculated
Tissue blood flow (fraction cardiac output)			
Fat	QFC	0.068	ICRP^22^
Brain	QBC	0.12	ICRP^22^
Heart	QHC	0.04	ICRP^22^
Skin	QSKC	0.05	ICRP^22^
Muscle	QMC	0.14	ICRP^22^
Liver	QLC	0.26	ICRP^22^
Rapidly perfused	QRC	0.19	Calculated
Slowly perfused	QSC	0.08	Calculated
Buccal cavity	QBUC	0.0215	Corley et al.^18^
Conducting airway	QCAC	0.025	Corley et al.^19^
Transitional airway	QTAC	0.007	Corley et al.^19^
Surface area (cm^2^)			
Buccal cavity	SABU	103.10	Corley et al.^18^
Conducting airway	SACA	199.50	Corley et al.^18^
Transitional airway	SATA	163.60	Corley et al.^18^
Pulmonary	SAPUL	540,000	ICRP^22^
Width epithelium (cm)			
Mucus	WMUC	0.0011	Corley et al.^18^
Buccal cavity	WTBU	0.0065	Corley et al.^18^
Conducting airway	WTCA	0.0065	Corley et al.^18^
Transitional airway	WTTA	0.0065	Corley et al.^18^
Pulmonary	WTPUL	0.000036	ICRP^22^
Width submucosa (cm)			
Buccal cavity	WXBU	1.50E−03	Corley et al.^18^
Conducting airway	WXCA	1.50E−03	Corley et al.^18^
Transitional airway	WXTA	1.50E−03	Corley et al.^18^

**Table 2:** Chemical specific parameters for the human nicotine PBPK model^4,5,23^.

Parameter	Label	Value	Source
**Partition coefficients**			
Nicotine			
Blood:Air	PB	10,000.00	Set to restrict exhalation
Lung	PLU	0.90	Satoskar et al.^23^
Fat	PF	0.80	Satoskar et al.^23^
Brain	PBR	3.00	Satoskar et al.^23^
Liver	PL	7.50	Satoskar et al.^23^
Heart	PH	1.60	Satoskar et al.^23^
Skin	PSK	1.50	Satoskar et al.^23^
Muscle	PM	1.50	Satoskar et al.^23^
Rapidly perfused	PR	7.50	Set to Liver
Slowly perfused	PS	1.50	Set to Muscle
Cotinine			
Liver	PML	2.00	Robinson et al.^4^
Muscle	PMM	1.50	Robinson et al.^4^
Rapidly perfused	PMR	1.50	Robinson et al.^4^
Slowly perfused	PMS	1.00	Robinson et al.^4^
Fat	PMF	0.50	Robinson et al.^4^
Metabolism (L/h/kg BW^0^^.75^)			
Nicotine in liver	CLMC	2.70	Robinson et al.^4^
Fraction to cotinine	FNC	0.80	Robinson et al.^4^
Cotinine in liver	CLLMC	0.14	Robinson et al.^4^
Urinary clearance (L/h/kg BW^0^^.75^)			
Nicotine	CLKC	0.42	Robinson et al.^4^
Cotinine	CLKMC	0.025	Robinson et al.^4^
Oral bioavailability	FA	0.67	Teeguarden et al.^5^
Oral absorption rate (h^−1^)	KA	1.34	Teeguarden et al.^5^
Pharmacodynamic parameters			
Concentration effect relationship	S	933.66	Teeguarden et al.^5^
First-order rate of loss of tolerance	KANT	1.6617	Teeguarden et al.^5^
Tolerance ‘‘concentration’’	CANT50	0.0152	Teeguarden et al.^5^

Tissue blood partition coefficients for nicotine were updated from those reported by Robinson and co-workers^4^, using the rat kinetic data reported by Satoskar and co-workers^23^, which had a broader range of tissues than those that were used in the study by Robinson et al.^4^. The partition coefficients for nicotine were calculated as the ratio of the concentration of nicotine in the tissue to the concentration of nicotine in the serum, at the last time-point reported in the study by Satoskar et al.^23^ (i.e., 25 min after intravenous dosing). The overall effect of this change was minimal to the time-course prediction of nicotine in plasma. The cotinine tissue:blood partitions were retained from Robinson et al.^4^.

The clearance of nicotine and cotinine occurs via renal metabolism and urinary excretion. Robinson and co-workers^4^ compiled multiple studies to determine rate constants for hepatic metabolism and urinary excretion. As this effort was primarily focused on extending nicotine intake to product specific contact, the constants reported by Robinson et al.^4^ were retained for this effort. In the Robinson nicotine PBPK model, nicotine metabolism is described as a total rate of hepatic clearance with a fraction (0.8) of the total being metabolized to cotinine.

The effective diffusivity and apparent permeability of nicotine in the BC and RT tissues were adopted from the study by Adrian and coworkers^17^.

### Software

Development and simulation with the nicotine PBPK model was conducted in R software, using several available packages to translate and compile the model. Specifically, the model was written in MCSim^24^, translated to C, and then compiled (Rtools, Ver. 3.3.0.1959) in R (Ver. 3.4.4). Integration was achieved using the deSolve package^25^ and the VODE algorithm. RStudio (Ver. 1.4.442) was used to provide a more efficient interface with R. The model code is included in the supplemental file.

## Results and discussion

**Figure 11:**
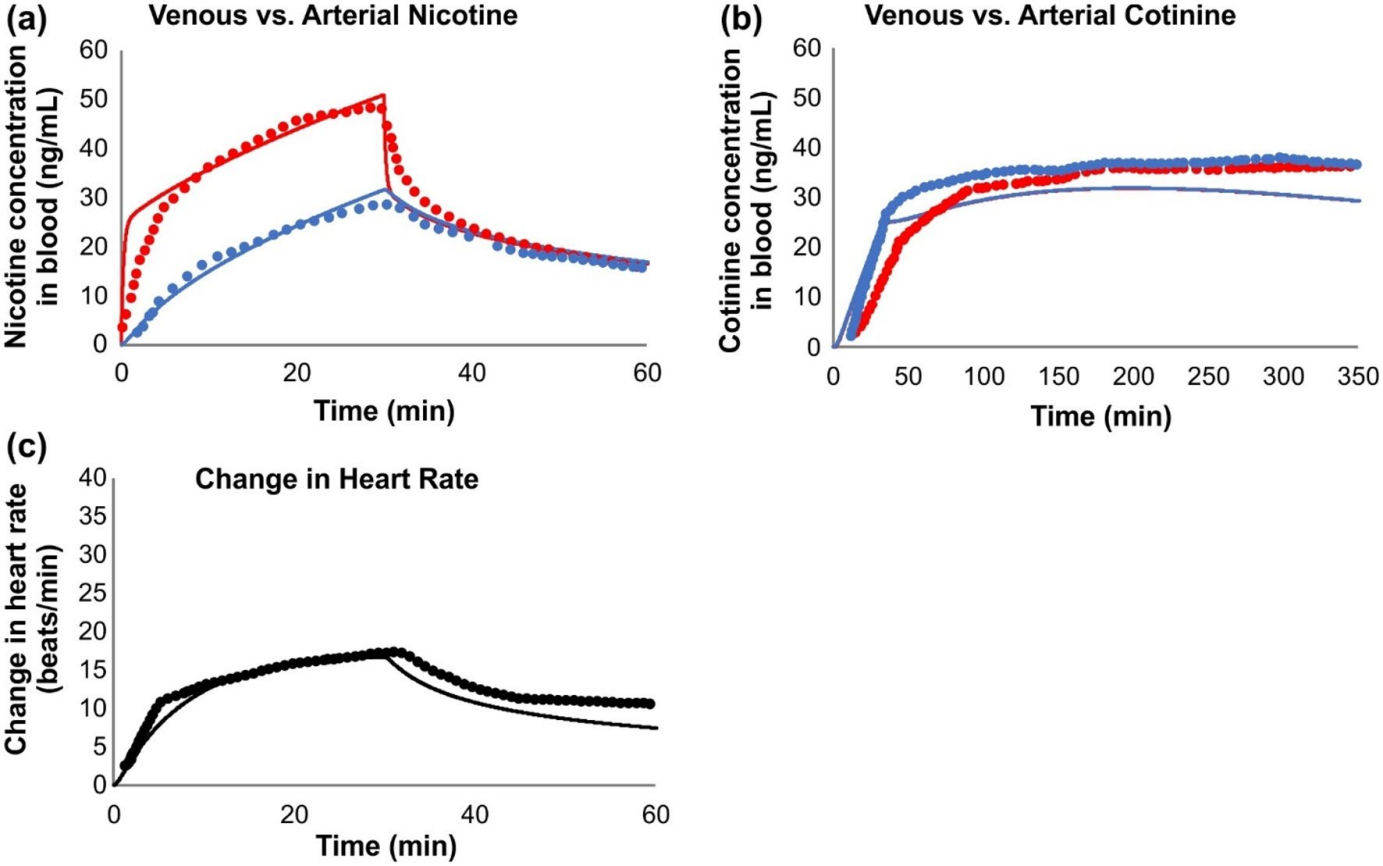
Simulation of the intravenous administration of nicotine in human^26^. Subjects (N = 22) were administered nicotine at a rate of 2 µg/kg/min for 30 min; dotted line represents data from study and solid lines represent simulation results. Red lines and dots correspond to arterial blood and blue lines and dots to venous blood.

To validate and evaluate our PBPK model’s capability for predicting nicotine distribution in the blood, we simulated five different exposure scenarios and compared the outcomes of modeling simulations with published results from clinical studies. As a simple example of the PBPK model application, we considered nicotine PK profile from intravenous administration of nicotine^26^. This example does not need the use of dosimetry and permeation models described earlier, as nicotine is directly injected into the arterial blood. The model prediction of the time-course arterial and venous plasma concentration for nicotine and cotinine during and after 30 min intravenous infusion of nicotine at a rate of 2 µg/kg/min^26^ are shown in Fig. 11a,b. Overall, the model provides excellent agreement with the venous and good agreement with the arterial nicotine concentrations reported in the study, although the model does tend to over-predict the initial appearance of nicotine in the arterial blood in the first few minutes. Given individual physiological and metabolic variability, the agreement for cotinine is good, with model predictions being within ± 20% of the reported time-course concentration curves. The model also captured the change in heart rate during and after exposure to nicotine (Fig. 11c), which was also in good agreement with measured outcomes. The overall excellent agreement between experimental and simulated results, speak to the validity of our predictions from the PBPK model for a well-defined exposure scenario. Next, we applied the model for four categories of products as described below.

### Conventional cigarettes

To further demonstrate validity of our PBPK model to accurately predict the time course of nicotine PK profile following smoking of conventional cigarettes, we simulated and compared model predictions against measured nicotine PK profiles from the four published studies described below. The studies had varying use conditions (e.g., single vs. repeated use) and products with different nicotine deliveries.

Picavet et al.^27^ reported the time-course venous concentration of nicotine in 28 subjects after a single use of a conventional cigarette. Venous plasma was collected at 2, 4, 6, 8, 10, 15, 30, 45, and 60 min, and at 3, 4, 6, 9, 12 and 24 h. In order to simulate the Picavet data, it was necessary to approximate the mass of nicotine inhaled from the conventional cigarette as this was not explicitly reported. The amount of inhaled nicotine per puff depends on many parameters including cigarette nicotine level and smoking topography such as puff volume, puff duration, mouth hold time, depth of inhalation and other variables. None of these were specified or controlled in the Piavet study^27^. Subjects were instructed to use their own brand with an ISO nicotine delivery of maximum 1 mg nicotine delivery per cigarette as labeled on the cigarette packs. Given all these uncertainties and the fact that users generally puff more intensely than smoking machine ISO conditions, we arbitrarily assumed 1.1 mg of nicotine was inhaled from each cigarette in 10 puffs over 9 min. This number is close to the nicotine delivery of a cigarette with a medium tar level under a smoking regime between ISO and Health Canada Intensive conditions.

In another study by Benowitz and coworkers^28^, 10 healthy men, 24 to 61 years of age, who were habitual cigarette smokers, were asked to smoke a single cigarette under controlled conditions. Subjects were directed to smoke 1 puff every 45 s, for a total of 12 puffs over 9 min. It was noted that the subjects’ own brand of cigarettes yielded an average of 1.1 mg of nicotine/stick with participants smoking approximately one and one third cigarette over the 9 min.

Two additional studies reporting venous plasma concentrations during and after repeated use of cigarettes were published by deBethizy and coworkers^29^ and Russell and coworkers^30^. deBethizy reported the venous plasma concentration for each of the 10 subjects, at various time points during and after smoking 7 cigarettes. The subjects were instructed to smoke at a rate of 1 cigarette every 30 min. The reported nicotine yield was 0.81 ± 0.2 mg per stick. Venous plasma concentrations were reported at 5.5, 7.5, 15, and 30 min after lighting cigarettes 1–6. Venous plasma concentrations were measured and reported at 5.5, 7.5, 10, 15, 20, 25, 30, 45, 60, 120, 180, and 240 min, after lighting of the seventh cigarette. The Russell study^31^ reported venous plasma nicotine concentrations in a single subject during and after cigarette use, at a rate of 1 cigarette/h over 7 h. Cigarettes were smoked over a 5 min period at 0, 7, 15, and 30 min after lighting each cigarette. In a second study design, a subject was tasked with smoking 3 cigarettes/h over 7 h. Samples were collected every 20 min starting with the lighting of the first cigarette.

For dosimetry estimates, for all the cigarette smoking scenarios modeled, it was assumed that approximately 95% of the inhaled nicotine mass reaches the alveolar region, where absorption would be instantaneous. The remaining inhaled mass was simulated as being deposited equally in the BC and the URT, with negligible transfer to the GI region. Figure 7c shows instantaneous transfer rate in the LRT. We assumed all the inhaled nicotine from cigarettes is absorbed with no exhaled amount.

**Figure 12:**
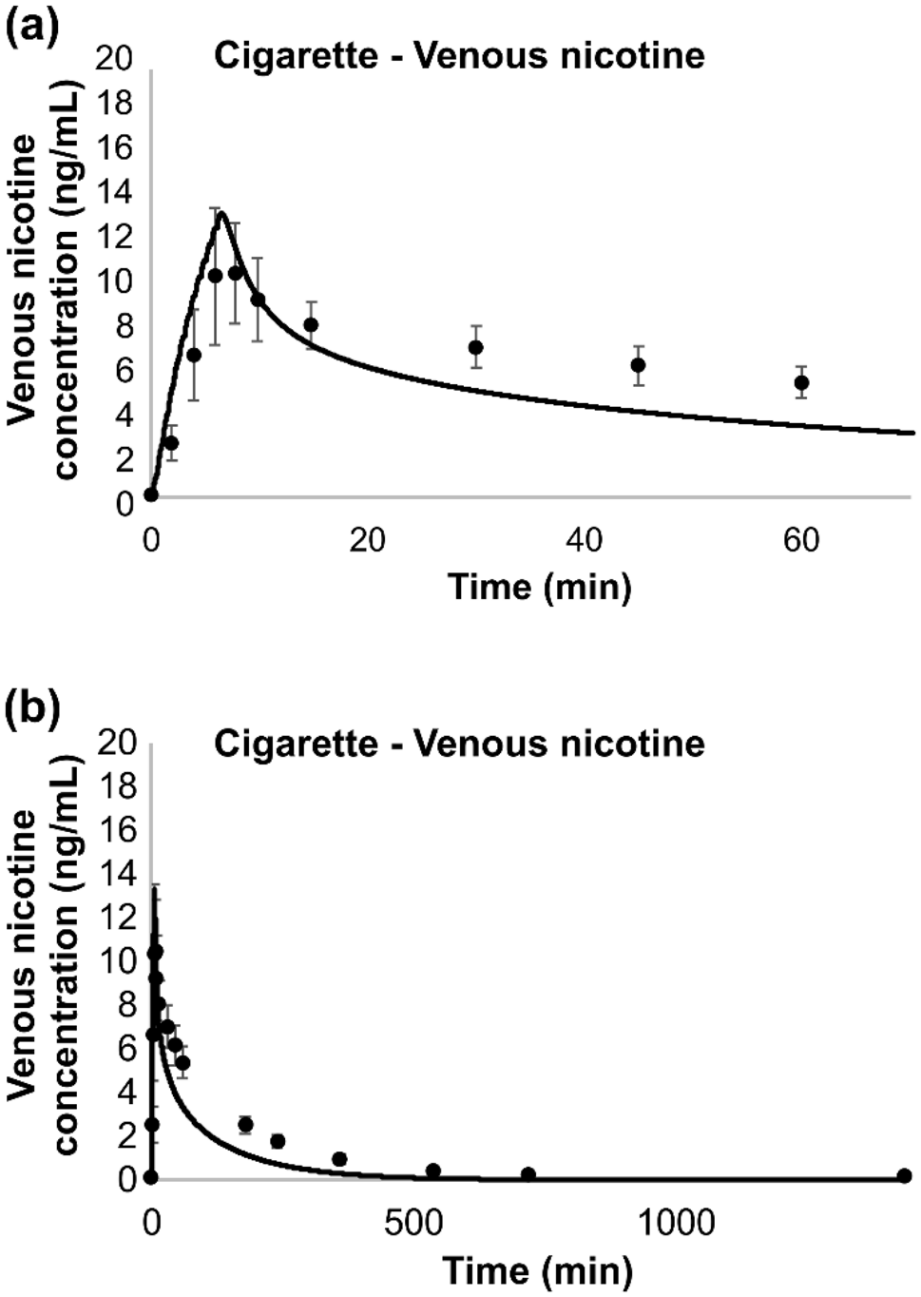
Model simulation of the single use of a conventional cigarette^27^: (**a**) expanded view of the first hour of (**b**) the overall simulation; points represent data from the study, along with associated standard deviations and solid lines represent simulation results.

**Figure 13:**
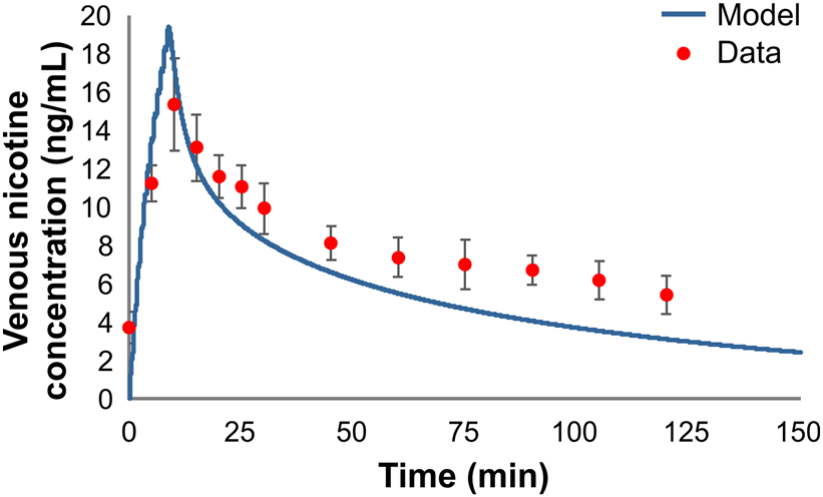
Simulation of the time-course kinetic data during and after a single use of a conventional cigarette^28^. Subjects were directed to take 1 puff every 45 s over a total of 9 min. The average yield was reported as 1.1 mg with subjects consuming 1.33 cigarettes over the 9 min. Points represent data from the study, along with error bars representing one standard deviation and solid lines represent simulation results.

**Figure 14:**
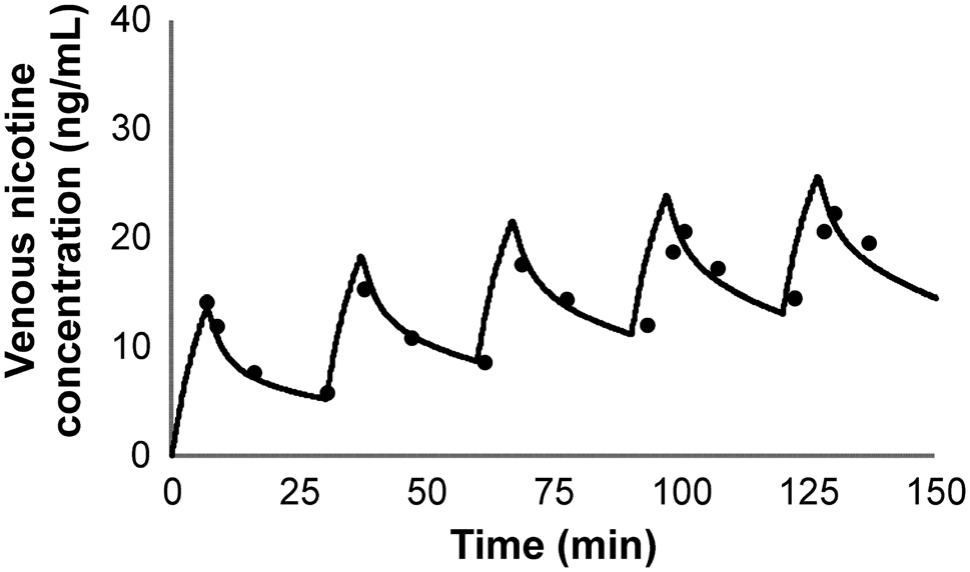
Simulation of the time-course kinetic data during and after repeated use of a conventional cigarette^29^. The subjects were allowed to smoke 1 cigarette every 30 min for a total of 7 cigarettes over the 7 h. Nicotine yield was reported as 0.81 ± 0.2 mg. Points represent data from the study and solid lines represent simulation results.

**Figure 15:**
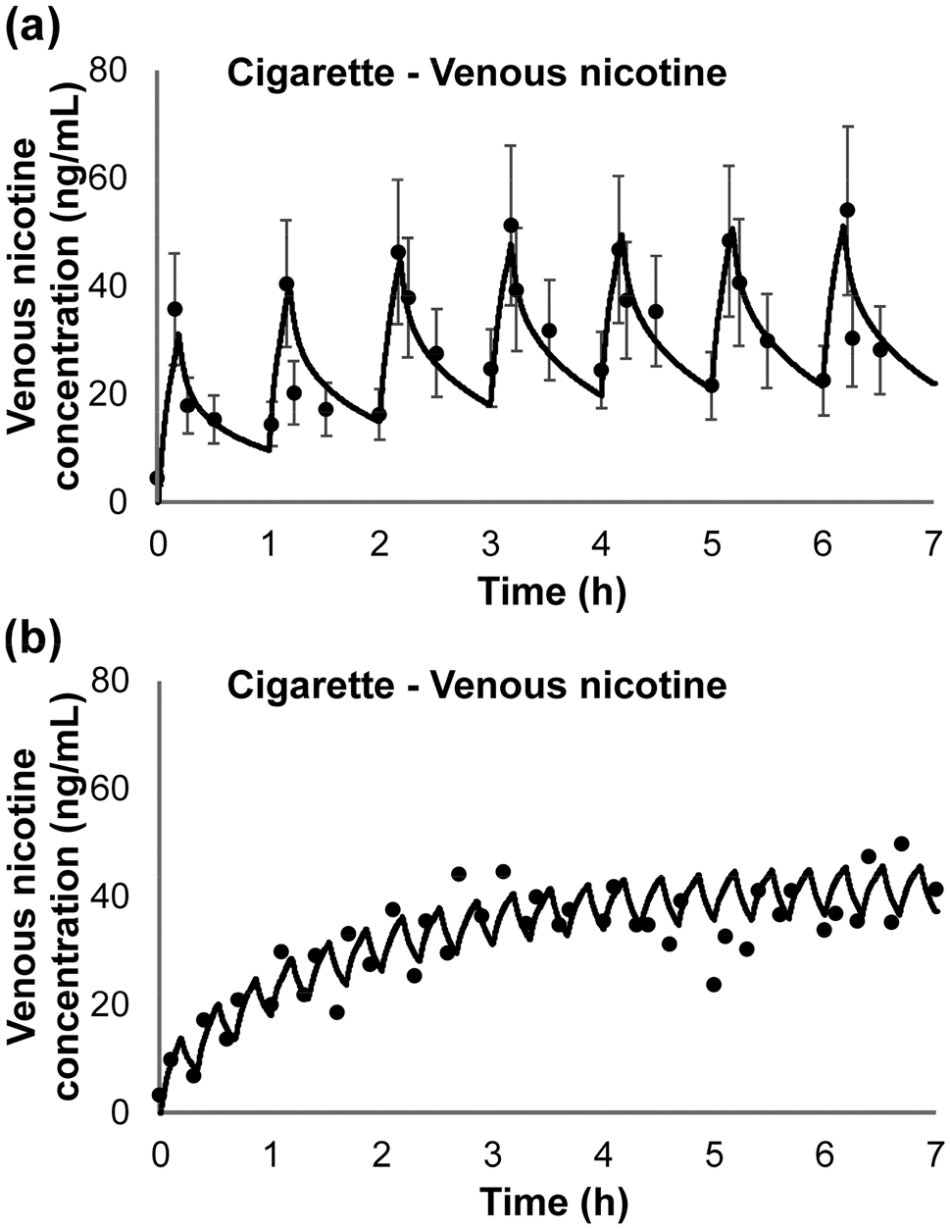
Simulation of the time-course kinetic data during and after repeated use of a conventional cigarette^31^. The subjects used (**a**) 1 cigarette/h over 7 h or (**b**) 3 cigarettes/h over 7 h. Points represent data from the study and solid lines represent simulation results.

The simulation of nicotine venous plasma concentration during and after single and repeated cigarette uses are shown in Figs. 12, 13, 14 and 15 for the four studies^27–30^, respectively. The model captured well the uptake and clearance of nicotine from cigarette, with model predictions generally being within one standard deviation of the data. For these simulations, we treated each repeated use, the same as the first use. That is, the mass of nicotine inhaled with each puff within a simulation was identical, regardless of puff volume, and only the estimated mass taken is varied across the study. This assumption held up for each of the two studies with repeated use in Fig. 14^29^ and Fig. 15^30^, where the model did an excellent job at capturing the overall pattern of nicotine in plasma even following repeated smoking of conventional cigarettes.

### ENDS products

The next example of the model application was focused on evaluating uptake of nicotine following use of ENDS products. For evaluating our PBPK model’s ability to predict the time course of nicotine following use of ENDS products, we simulated and compared our PK curve predictions against measured nicotine PK profiles from a study conducted by Lopez and coworkers^32^. In that study, the researchers measured and reported data over two consecutive uses of an ENDS product (eGo device; 3.3 V, 1000 mAh battery with a 1.5 Ω, dual-coil, 510-style cartomizer). Each of the 16 ENDS-naïve cigarette smoker study participants who completed the study (defined in their publication as healthy, aged 18–55 years, used at least 15 cigarettes daily, and used an e-cigarette less than 5 instances in their lifetime) participated in four randomized sessions. Depending on the randomization sequence, a participant was provided with a product containing e-liquid with either 0, 8, 18, or 36 mg/mL of nicotine, in each of their four sessions. In each session, the study participants, were asked to take a total of 10 puffs with a 30 s interval per use (1 h interval between the two uses). No data were provided on the amount of nicotine intake from the device.

Since the amount of nicotine inhaled per puff for the three nicotine strengths is not specified in the publication^32^, we needed to calculate those from other information provided in the publication. The amount of aerosol generated per puff depends on the power input and puff duration. Although this can vary among different devices, we follow a semi-empirical approach. In general, of the total amount of electrical energy supplied to the device, a portion is used for heating the system. The rest is used to heat and vaporize the e-liquid, which is generally a mixture of propylene glycol, glycerine, water, and nicotine. Using thermodynamics values, the total amount of energy (sensible and latent) needed to vaporize 1 mg of an e- liquid mixture is approximately 1.5 J. This is the estimated energy needed to heat the e-liquid from the room temperature to approximately 275 °C. Lopez et al.^32^ provided the battery voltage (3.3 V) and the heater resistance (1.5 Ω), and reported puff durations ranging from 2.2 to 2.9 s. The power input is calculated to be 7.26 W, and the total energy inputs were 21, 20.38, and 16 J for the 8, 18, and 36 mg/mL e-liquids, respectively. Out of this energy, 8.7 J is used to heat up the system, which corresponds to the energy consumed before any aerosol is formed^33^. Subtracting this energy from total energy and dividing by 1.5 (J/mg), we estimated the aerosol mass per puff, and the corresponding nicotine inhaled per puff as 0.066, 0.14, and 0.176 mg/puff for the 8, 18, and 36 mg/mL nicotine levels, respectively. Assuming 90% of inhaled aerosol is absorbed with 10% exhaled, the final nicotine dose inputs to the PBPK model used were 0.06, 0.126 and 0.158 mg/puff of nicotine, in the order of increasing nicotine level in the e-liquid.

Following the same procedure introduced for the dosimetry model, the distribution of absorption in the RT of 10% in BC, 15% in URT and 75% in LRT, was used for modeling this scenario. For this distribution, we also had to calculate the vapor-particle partitioning of nicotine. Since no information on the pH of the e-liquid was given in the paper, an activity coefficient of 10 was used to correct the Raoult’s law for nicotine partitioning between liquid and vapor phase over liquid mixture. Such a large activity coefficient is not unusual for low molar fraction of nicotine in the liquid mixture. No transfer to GI tract was assumed, because only 10% of nicotine is absorbed orally and the use duration is only 5 min per session.

Next, we employed the permeation model to estimate deposition location specific transfer rates of nicotine to the circulation system (similar to Fig. 7a–c, but different ratios) for each region of the airway; BC, URT, and LRT, respectively. They are presented separately as these transfers occur on significantly different timescales in these three regions. The blood flow rates vary in different regions and as previously discussed they are treated differently in the PBPK model.

**Figure 16:**
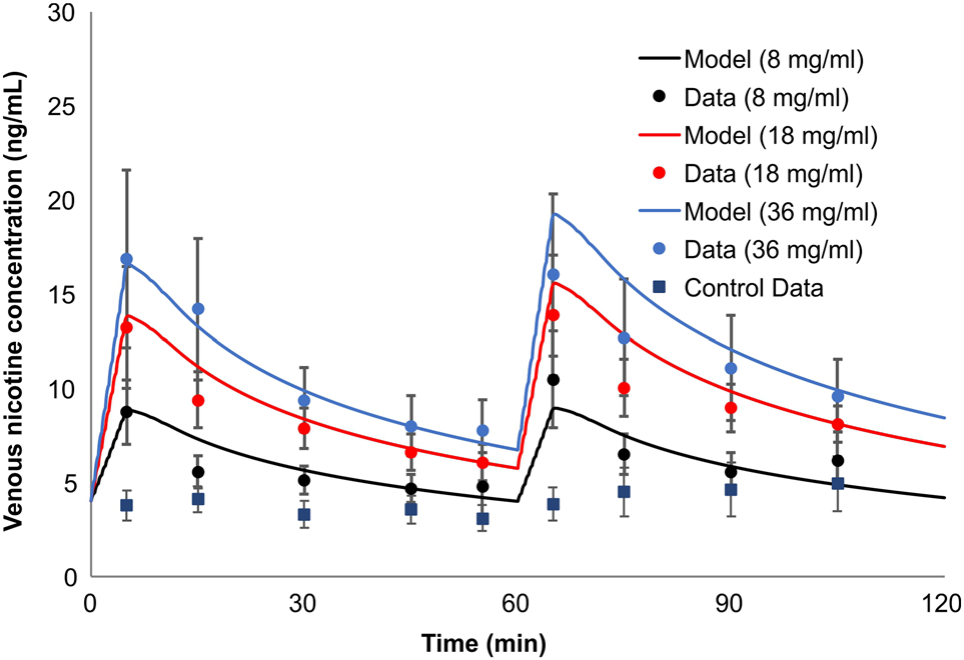
Simulation of the nicotine venous plasma time-course in 16 ENDS-naïve cigarette smokers after two uses (1 h interval) of an ENDS device reported^32^. The nicotine content of the e-liquids was reported as 8, 18, and 36 mg/mL. Subjects were asked to take 10 puffs with 30 s puff intervals. Error bars represent one standard deviation.

A comparison of the predicted venous nicotine plasma time-course to the experimental data is presented in Fig. 16. The error bars in the figure represent one standard deviation. In this case, however, the assumption of identical intake across two uses (60 min apart) was not as fruitful as it was with the simulations for cigarette smoking. Our PBPK model provided excellent agreement with the first use, but slightly over predicted the time-course plasma for higher nicotine concentration e-liquids, upon the second use. The mean concentrations from the model predictions all lie within one standard deviation of the reported data. Given that the uptake also depends on the depth of inhalation, which is not specified in the study, a possible explanation may be that subjects altered their intake rate by reducing their depth of inhalation for e-liquid with high nicotine concentration. Lower depth of inhalation results in lower absorption for this case and lower measured nicotine concentration in plasma. Based on sensitivity analysis that we performed with our model, depth of inhalation would have a significant impact on the overall outcome of the simulation. In general, a shallow depth of inhalation results in less aerosol reaching the LRT, thus less uptake and more exhale. In addition, the subjects were described as naïve users of ENDS products, who were predominantly smokers of conventional cigarettes, and this could also have played a role in the altered intake with the second use. Other explanations related to the topography changes that may affect the actual PK profile are also feasible and require further investigation.

### Vapor inhaler

The next example is related to a vapor inhaler. For this, we simulated the study of Liu and coworkers^34^ and compared our predicted results with the outcomes reported in their clinical study. In their study^34^, nicotine venous plasma concentrations were measured, during and after use of the NICOTROL nicotine inhaler. Individual subjects were asked to take 80 puffs (2 s puff every 15 s) over a 20 min period. Based on the manufacturer’s information^35^, the total mass inhaled over 80 puffs was 2 mg. From the vapor absorption-based dosimetry model described earlier, it was estimated that 95% of nicotine is absorbed in the BC and 5% in the URT airway; 70% of the orally absorbed nicotine was assumed to transfer to GI tract, as the 20 min use duration included multiple instances of swallowing. The rate of nicotine transfer to the plasma for vapor inhaler was estimated using the permeation model, and is presented in Fig. 8.

**Figure 17:**
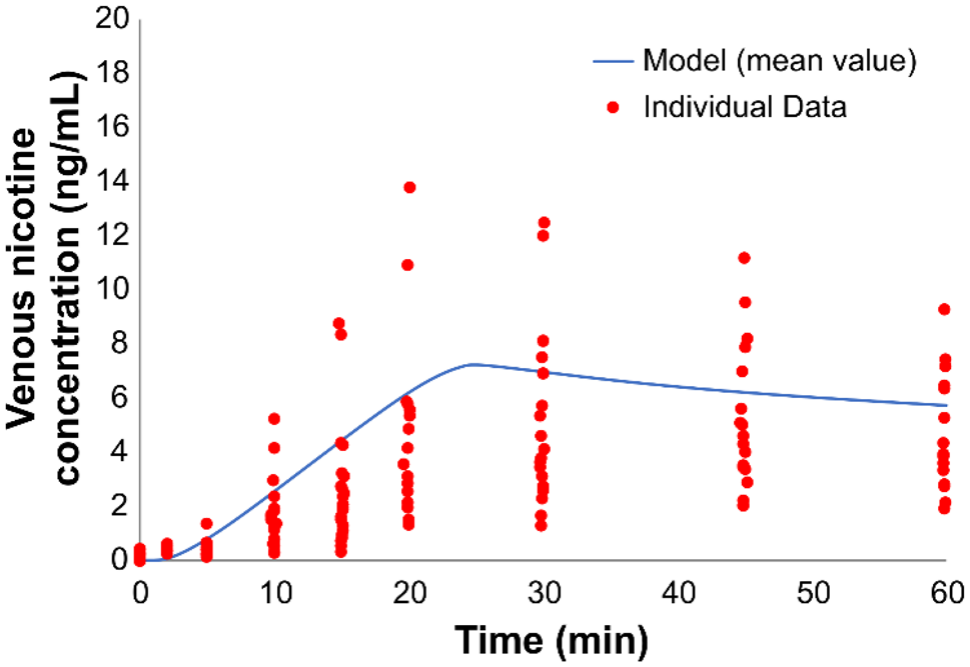
Simulation of nicotine venous plasma concentration during and after use of a nicotine inhaler (NICOTROL). Data are for individual subjects taking 80 puffs (2 s puff every 15 s) over a 20 min period and are represented as dots. Solid line represents the simulation result. The total mass inhaled was 2 mg with an estimated 95% deposited in the BC and 5% in URT.

Figure 17 compares the simulated PK profile following use of a vapor inhaler and compares the model predictions to experimental data^36^ PK profiles. Overall, the PBPK model well captured the slow and muted rise to maximum concentration which occurred near the end of exposure (i.e., 20 min), which was driven by a larger proportion of the inhaled mass being transferred to the GI tract following oral absorption. Good agreement between the model predictions and mean of the experimental data further affirms the validity of applying our PBPK to predict nicotine PK from another type of nicotine containing product.

### Smokeless tobacco

Among the different product uses, this was the most challenging product use to simulate. The mass of nicotine in the product is extracted with saliva in the BC, where it is either absorbed locally, swallowed to the GI tract, or removed via spittle through expectoration. To evaluate the PBPK model’s viability to predict nicotine PK in users of oral tobacco products, we simulated the study of Digard et al.^37^, which reported the venous plasma time-course of nicotine during and after the use of different smokeless tobacco formats; subjects were asked to keep the loose snus (10.79 or 27.09 mg nicotine/plug) or pouched snus (10.72 or 14.67 mg nicotine/pouch), in place for the first hour of the study. The authors also reported the nicotine mass remaining in the products after use, which allowed the estimation of the nicotine extracted over the use period. The amount of nicotine available for absorption by the body is the difference between the pre-use and post-use nicotine content. However, for the PBPK model, in addition to the amount extracted, the time profile of nicotine release over the 60 min use period is needed. Following the procedure described for smokeless tobacco (Figs. 4, 5), the rate of nicotine release in the BC was approximated as exponentially decreasing over time, m = m_0_ e^−bt^, where m_0_ is the rate of nicotine release at time 0 (mg/min), b is the rate constant (1/min) and t is the time in minutes. Values of m_0_ and b are selected such that the total mass released during the usage period matched the measure values reported by Digard et al.^37^, and are shown in Table 3. For smokeless tobacco, 100% of the nicotine absorption is assumed to take place in the BC, with no swallowing. The relative transfer rate of nicotine from the BC to the plasma, corresponding to nicotine release/dissolution presented in Fig. 6, was estimated using the previously described permeation model. The predictions for relative transfer rate at which nicotine is removed from the BC to the plasma is shown in Fig. 9.

**Table 3:** Constants for the rate of nicotine release in the BC during the 60 min use time matching data of Digard et al.^37^: m = m_0_ e^−bt^.

Product	m_0_ (mg/min)	b (1/s)
Loose snus (10.79 mg/mL)	0.0678	0.006
Loose snus (27.09 mg/mL)	0.120	0.004
Pouched snus (10.72 mg/mL)	0.065	0.006
Pouched snus (14.67 mg/mL)	0.0879	0.006

*m*_*0*_ rate of nicotine release at time 0, *b* rate constant, *t* time.

**Figure 18:**
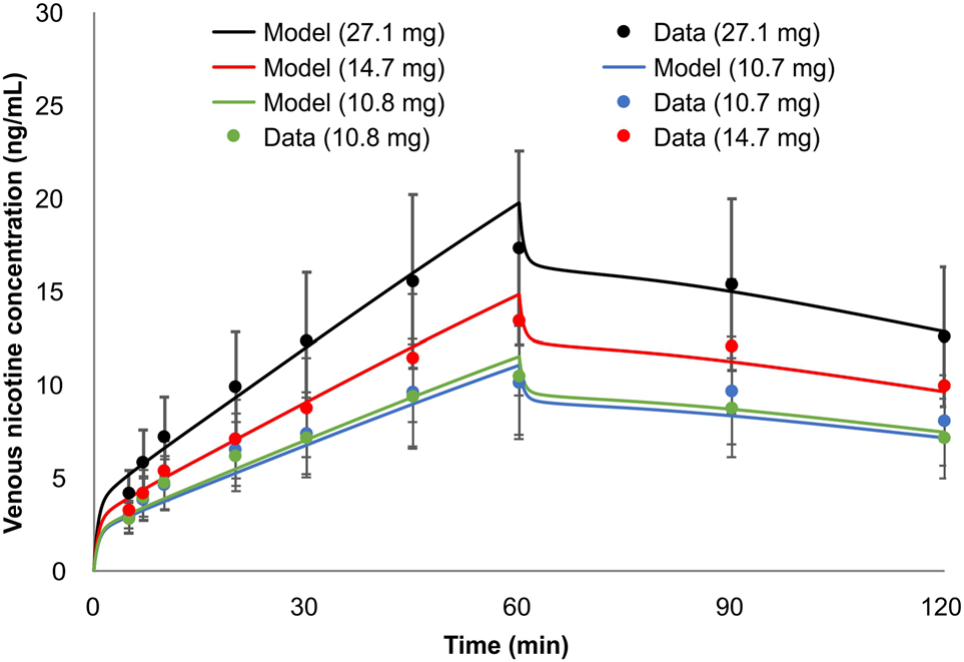
Simulation of the nicotine venous plasma concentration in 20 subjects during and after a single use of MST. Four separate uses were administered including two loose snus (27.1 mg/plug, black and 10.8 mg/plug, green) and two pouched snus (14.7 mg/pouch, red and 10.7 mg/pouch, blue). Plugs remained in place for the first 60 min of the study^37^. Error bars represent one standard deviation.

As with the previous simulations, the comparison of model predicted and measured nicotine plasma concentrations shown in Fig. 18, demonstrate that predictions were within one standard deviation of reported data. The mean predicted nicotine levels were all within the individual variation of PK data for study participants in the study^37^, further affirming our PBPK model’s prediction capabilities.

## Conclusions

We have successfully developed a physiological based PK model that can be used for characterizing nicotine PK from intravenous dosing and use of four different types of nicotine containing products; including those that deliver nicotine orally or produce nicotine containing inhalable vapors and/or aerosols.

The overall goal of this effort was to extend the previously published nicotine PBPK models to allow simulation of the product-specific uptake of nicotine across a wide range of nicotine-containing products. The inclusion of dosimetry and permeation models that incorporated a description of diffusion across the mucus and epithelium in the BC and RT, provided a basis on which we could address differences in product deposition patterns or release, as well as transfer between compartments (e.g., swallowing of material in BC). This allowed our effort to focus on the impact of regional specific deposition on the PK profile of the venous plasma concentration of nicotine, when necessary data may not be available to provide exact simulation of the inhalation pattern (i.e., puff volume and concentration of nicotine in the smoke/aerosol stream).

The nicotine PBPK model presented here is a step forward in the development of modeling of nicotine PK in humans and provides a quantitative basis for assessing changes in product design on absorption, when information on the RT deposition patterns can be estimated using secondary dosimetry and permeation models. The assumptions incorporated into the models, such as assuming identical use patterns across all uses in repeated use studies, the need for estimating fractions transferred to the GI tract, estimating the rate at which a user may spit out an oral product, etc. could translate into model limitations. However, the model successfully simulates nicotine PK curves, which were found to be in good agreement with those generated from clinical studies, for a variety of inhalable and oral nicotine delivery products. A secondary goal of this effort was to provide a base model which may be expanded to address exposure to other constituents in the product-use stream including chemicals of concern during product-risk assessments. The assumptions made in this effort provide a basis to move forward with addressing route-specific exposure to these compounds, however for new categories of nicotine delivery products, product-specific PK data may initially be required for model evaluation. The model can be further improved if more experimental data on the physicochemical properties that influence the nicotine release and vapor particle partitioning become available.

The PBPK model presented here is highly flexible and provides a rapid screening tool to perform a broad spectrum of sensitivity analysis that allow for characterizing nicotine PK profile from (1) using single and/or multiple different products; (2) understanding variability in PK results across subjects, driven by differences in use topography and/or physiology (e.g., body weight); and (3) different nicotine delivery of products of the same category.

## Supplementary Information

Supplementary Information.
