# Author Correction: A comprehensive physiologically based pharmacokinetic (PBPK) model for nicotine in humans from using nicotine-containing products with different routes of exposure

**DOI:** 10.1038/s41598-022-06693-8

**Published:** 2022-02-08

**Authors:** Ali A. Rostami, Jerry L. Campbell, Yezdi B. Pithawalla, Hamideh Pourhashem, Raheema S. Muhammad-Kah, Mohamadi A. Sarkar, Jianmin Liu, Willie J. McKinney, Robinan Gentry, Maria Gogova

**Affiliations:** 1 Center for Research and Technology, Altria Client Services LLC, 601 East Jackson Street, Richmond, VA 23235 USA; 2 Ramboll, 3214 Charles B. Root Wynd, Suite 130, Raleigh, NC 27612 USA; 3 Ramboll, 3107 Armand Street, Monroe, LA 71201 USA

Correction to: *Scientific Reports* 10.1038/s41598-022-05108-y, published online 20 January 2022

The original version of this Article contained an error in Reference 8, which was incorrectly given as:

Kovar, L. *et al.* Physiologically-based pharmacokinetic (PBPK) modeling of buprenorphine in adults, children and preterm neonates. *Pharmaceutics* **12**(6), 578 (2020).

The correct reference is listed below:

Kovar, L. *et al.* Comprehensive Parent–Metabolite PBPK/PD Modeling Insights into Nicotine Replacement Therapy Strategies. *Clin Pharmacokinet* **59,** 1119–1134 (2020). https://doi.org/10.1007/s40262-020-00880-4

The original Article has been corrected.

